# Hospital Sites in London

**Published:** 1903-05-09

**Authors:** 


### Editor's Letter-Box.

### HOSPITAL SITES IN LONDON.

A Correspondent writes: Since the question of removing certain of the great London hospitals from their present sites to neighbourhoods which are now but ill served with medical charities has been definitely raised, it may not be without use to review the relative needs of two of the hospitals whose names have come somewhat prominently before the public—St. George's and King's College Hospital.

So far as the physical conditions of the site are concerned, St. George's occupies a position unequalled in London. With Hyde Park on the north and the large open space with the Green Park beyond on the east, it enjoys on these two sides an open space equalled by few, if any, hospitals of its size anywhere. On the south Grosvenor Place is 100 feet wide at its narrowest point, and it is only on the West side that the building can be said in any degree to be built in.

In plan the building certainly leaves much to be desired. Planned in the form of an H, with one wing extended, the centre part contains wards with windows on one side only and with a central corridor. The sanitary offices also are by no means up to date. Large sums of money have been spent in recent years in enlargements and improvements, notwithstanding which it was stated at a recent meeting that the operation theatre, a recently constructed and extravagantly fitted room, was unsuitable for its purpose. It ought to be possible to remedy the most important of these defects at no very excessive outlay; and if this be conceded there seems little justification for removing the hospital on the score of defective site or buildings.

King's College Hospital is very differently situated. Surrounded by streets and comparatively high buildings, the hospital has not a square inch of ground on which to expand. The building itself is probably the worst planned hospital of its size in London. The wards are of the double type, similar to those at St. Bartholomew's, but lacking the free air space which must be of such inestimable value to the latter; some wards there are 36 feet long with windows at one end only, and the sanitary offices are cramped, inadequate, and with little possibility of improvement. The nurses' quarters, though much improved of late years, are not by any means up to the standard of modern times. Finally, the out-patient department is on two floors and is wholly inadequate to present-day needs. Improvements and additional accommodation are urgently needed in almost every department of the hospital, and there is no space to carry them out efficiently.

It would seem, therefore, that on an impartial comparison of their respective needs the case for moving King's College Hospital is many times stronger and more urgent than that for St. George's.

[*** Our correspondent has evidently not recently visited St. George's Hospital. If he had done so he would realise that it contains from 100 to 140 bad beds, which ought not to be retained in the wards; that it is impossible to reconstruct the present buildings so as to afford adequate bath and lavatory accommodation; and that if the present site is to be retained, assuming that St. George's Hospital is ever to be brought up to the requirements of a modern hospital, it will probably be necessary to pull down the whole of the existing buildings and to rebuild entirely. These are the reasons which make the problem of St. George's Hospital so important and pressing. It will not be easy for the Governors to arrive at a definite decision one way or the other; but we hope, whatever that decision be, it will be based upon an intelligent apprehension of modern requirements, and the claims of St. George's Hospital to take its place as one of the foremost medical schools of the British Empire.—Ed. "The Hospital."]

